# What Is Cholera?

**Published:** 1866-01

**Authors:** 


### HALL'S JOURNAL OF HEALTH.

VOL. 13. JANUARY, 1866. No. I.

### WHAT IS CHOLERA?

Cholera is the exaggeration of intestinal vermicular motion

This definition, explained in language less professional, would do more good than all the popular recipes for the cure of Cholera ever published, because it expresses the inherent nature of Cholera and suggests the principles of cure, in its early stage, to the most unreflecting mind.

The public is none the better, or wiser, or safer, for one of all the ten thousand "cures" for Cholera proclaimed in the public prints, with a confidence which itself is a sufficient guarantee that however well-informed the authors may be in other matters, as regards Cholera itself they are criminally ignorant; for no man has a right to address the public on any subject connected with its general health unless he understands that subject in its broadest sense, practically as well as theoretically.

As Cholera has become a general and perhaps, at least for the present,* a permanent disease of the country, and at this time is more or less prevalent in every State of the Union—and one, too, which may at any hour sweep any one of us into the grave—it belongs to our safety to understand its nature for ourselves, and do what we may to spread the knowledge among those around us.

A "live" cheese or a cup of fishing worms may give an idea of the motion of the intestines in ordinary health. The human gut is a hollow, flexible tube, between thirty and forty feet long; but, in order to be contained within the body, it is, to save space, arranged as a sailor would a coil of rope, forever moving in health—moving too much in some diseases—too little in others. To regulate this motion is the first object of the physician in every disease. In head-aches, bilious affections, costiveness and the like, this great coiled-up intestine,

* 1854.

usually called "the bowels," is "torpid," and medicines are given to wake it up, and what does that cures the man. Costiveness is the foundation—that is, one of the first beginnings—or it is the attendant of every disease known to man, in some stage or other of its progress. But the human body is made in such a manner, that a single step cannot be taken without tending to move the intestines; thus it is, in the main, that those who move about on their feet a great deal have the least sickness,—and, on the other hand, those who sit a great deal, and hence move about but little, never have sound health; it is an impossibility—it is a rule to which I have never known an exception.

Cholera being a disease in which the bowels move too much, the object should be to lessen that motion; and, as every step a man takes, increases intestinal motion, the very first thing to be done in a case of cholera is to secure quietude. It requires but a small amount of intelligence to put these ideas together, and if they could only be burnt in on every heart, this fearful scourge would be robbed of myriads of its victims.

There can be no cure of Cholera without quietude—the quietude of lying on the back.

The physician who understands his calling is always on the look-out for the instincts of nature; and he who follows them most, and interferes with them least, is the one who is oftenest successful. They are worth more to him than all the rigmarole stories which real or imaginary invalids pour in upon the physician's ear with such facile volubility. If, for example, a physician is called to a speechless patient—a stranger, about whom no one can give any information—he knows, if the breathing is long, heavy and measured, that the brain is in danger; if he breathes quick from the upper part of the chest, the abdomen needs attention; or if the abdomen itself mainly moves in respiration, the lungs are suffering. In violent cases of inflammation of the bowels, the patient shrinks involuntarily from any approach to that part of his person. These are the instincts of nature, and are invaluable guides in the treatment of disease.

Apply this principle to cholera, or even common diarrhœa, when the bowels do not act more than three or four times a day; the patient feels such an unwillingness to motion that he even rises from his seat with the most unconquerable reluctance; and when he has, from any cause, been moving about considerably, the first moment of taking a comfortable seat is perfectly delicious, and he feels as if he could almost stay there always. The whole animal creation is subject to disease, and the fewest number, comparatively speaking, die of sickness; instinct is their only physician.

Perfect quietude, then, on the back, is the first, the imperative, the essential step towards the cure of any case of cholera. To this art may lend her aid towards making that quietude more perfect, by binding a cloth around the belly pretty firmly. This acts beneficially in diminishing the room within the abdomen for motion; a man may be so pressed in a crowd, as not to be able to stir. This bandage should be about a foot broad, and long enough to be doubled over the belly; pieces of tape should be sewn to one end of the flannel, and a corresponding number to another part, being safer and more effective fastenings than pins. If this cloth is of stout woollen flannel, it has two additional advantages—its roughness irritates the skin and draws the blood to the surface from the interior, and by its warmth retains that blood there; thus preventing that cold, clammy condition of the skin which takes place in the last stages of cholera. Facts confirm this. When the Asiatic scourge first broke out among the German soldiery, immense numbers perished; but an imperative order was issued, in the hottest weather, that each soldier wear a stout woollen flannel abdominal compress, and immediately the fatality diminished more than fifty per cent. If the reader will try it, even in cases of common looseness of bowels, he will generally find the most grateful and instantaneous relief.

The second indication of instinct is to quench the thirst. When the disease now called Cholera first made its appearance in the United States, in 1832, it was generally believed that the drinking of cold water, soon after calomel was taken, would certainly cause salivation; and, as calomel was usually given, cold water was strictly interdicted. Some of the most heart-rending appeals I have ever noticed were for water, water! I have seen the patient with deathly eagerness mouthe the finger-ends of the nurse, for the sake of the drop or two of cold water there while washing the face. There are two ways of quenching this thirst, cold water and ice. Cold water often causes a sense of fulness or oppression, and not always satisfying; at other times the stomach is so very irritable, that it is ejected in a moment. Ice does not give that unpleasant fulness, nor does it increase the thirst, as cold water sometimes does, while the quantity required is very much reduced.

### A CASE.

About a year ago, I was violently attacked with cholera symptoms in a rail-car. The prominent symptoms were a continuous looseness of the most exhausting character, a deathly faintness and sickness, a drenching perspiration, an overpowering debility, a feeling as if the whole intestines were wrung together with strong hands, as washerwomen wring out clothing. Not being willing to take medicine, at least for a while, and no ice being presently obtainable, at the first stopping-place I ate ice-cream, or rather endeavored to swallow it before it could melt. I ate large quantities of it continually, until the thirst was entirely abated. The bowels acted but once or twice after I began to use it, I fell asleep, and next morning was at my office, as usual, although I was feeble for some days. This may not have been an actual case of Asiatic Cholera, although it was prevalent in the city at that time; but it was sufficiently near it to require some attention, and this is the main object of this article, to wit: attention to the first symptoms of Cholera when it prevails.

According to my experience, there is only one objection to the ice-cream treatment, and that is, you must swallow it without tasting how good it is; it must be conveyed into the stomach as near an icy state as possible.

The second step, then, in the treatment of an attack of Cholera, is to quench the thirst by keeping a plate of ice beside you, broken up in small pieces, so that they may be swallowed whole, as far as practicable; keep on chewing and swallowing the ice until the thirst is most perfectly satisfied.

### PRACTICAL RESULTS.

The first step, then, to be taken where Cholera prevails and its symptoms are present, is:

To lie down on a bed.

2d. Bind the abdomen tightly with woollen flannel.

3d. Swallow pellets of ice to the fullest extent practicable.

4th. Send for an established, resident, regular physician. Touch not an atom of the thousand things proposed by brains as "simple" as the remedies are represented to be, but wait quietly and patiently until the arrival of your medical attendant.

But many of my readers may be in a condition, by distance or otherwise, where it is not possible to obtain a physician for several hours, and where such a delay might prove fatal. Under such circumstances, obtain ten grains of calomel and make it into a pill with a few drops of gum water; dry it a little by the fire or in the sun and swallow it down. If the passages do not cease within two hours, then swallow two more of such pills, and continue to swallow two more at the end of each two hours until the bowels cease to give their light-colored passages, or until the physician arrives.

### WHY?

In many bad cases of Cholera, the stomach will retain nothing fluid or solid, cold water itself being instantly returned. A calomel pill is almost as heavy as a bullet; it sinks instantly to the bottom of the stomach, and no power of vomiting can return it. It would answer just as well to swallow it in powder; but the same medium which would hold it in suspension while going down, would do the same while coming up.

### THE FIRST OBJECT

Of a calomel pill in Cholera, is to stop the passages from the bowels. This is usually done within two hours; but if not, give two next time, on the principle if a certain force does not knock a man down the first time, the same force will not do it the second. Hence, to make the thing sure, and to lose no time—for time is not money here, but life—give a double portion. Not one time in twenty will it be necessary to give the second dose—not one time in a thousand the third. But as soon as your physician comes, tell him precisely what you have done, what its apparent effects, and then submit yourself implicitly to his direction.

When the calomel treatment is effectual, it arrests the passages within two hours; and in any time from four to twelve hours after being taken, it affects the bowels actively, and the passages are changed from a watery thinness to a mushy thickness or consistency, and instead of being the color of rice-water, or of a milk and water mixture, they are brown or yellow, or green or dark, or black as ink, according to the violence of the attack. Never take anything to "work off" calomel, if there is any passage within ten hours after it is taken; but if there is no passage from the bowels within ten, or at most twelve hours after taking calomel, then take an injection of common water, cool or tepid. Eating ice or drinking cold water after a dose of calomel, facilitates its operation, and never can have any effect whatever towards causing salivation; that is caused by there being no action from the bowels, as a consequence of the calomel, sooner than ten or twelve hours after it has been swallowed.

WHAT ARE THE FACTS?

I have been between two and three years in the midst of prevalent Cholera, continuously, winter and summer, the deaths being from two to two hundred a day. In all that time I had no attack, never missed a meal for the want of appetite to eat, ate in moderation whatever I liked and could get, and lived in a plain, regular, quiet way. During this time I had repeated occasions to travel one or two thousand miles, or more, in steamboats on the Mississippi, with the thermometer among the eighties in the shade and over a hundred on the deck, with from one to three hundred passengers on board, many of whom were German emigrants, huddled up around the boilers of a Western steamer—boatmen, Dutchmen and negroes, men, women and children, pigs and puppies, hogs and horses, living in illustrated equality. These persons came aboard from a hot and dusty levee, crammed with decayed apples, rotting oranges, bad oysters, and worse whisky; and almost invariably the report of the first morning out would be Cholera among the deck passengers, and the next thing, Is there a physician on board? Sometimes I was the only one; at others there were several, and we would divide. Practice of this kind is always gratuitous, and is attended with much personal labor, discomfort and exposure. On the last occasion of this kind I treated eighteen cases, all of whom were getting well, apparently, when landed along the river at their various homes, my destination being usually as far as the boat would go. There were only two deaths—one during the first night, before it was known that the cholera was aboard, the other occurred just as the boat was landing at the young man's home; how anxious he was to reach that home alive, no pen can ever portray. I did nothing for him. Before I knew he was sick, he was in the hands of a stranger who came aboard, and who had a remedy which was never known to fail. During the voyage, my patients slept around the steamboilers in midsummer, or on the outer guards, exposed to the rain which several times beat in upon them and their bedding; being every night just at the water's edge, and no protection against its dampness, nor against the sun in the heat of the day. And yet with these unfavorable attendants, not one of the eighteen died on board the "Belle Key," in her six days' journey. In all these cases the treatment was uniform: quiet, ice, and calomel pills, which last I was accustomed to carry with me. Some of them had been made five years, but lost none of their efficacy. Whether it was the ice, or the quiet, or the pills, or faithful nature which kept these persons from dying, I do not pretend to say; I merely state the doings and the result.

My own views as to the cure of Cholera, *as far as I have seen*, are, that when calomel fails to cure it, every thing else will fail, and that it will cure every curable case.

PREMONITORY SYMPTOMS OF CHOLERA.

The cure of this scourge depends upon the earliness with which the means are used. It can be said with less limitation than of all other diseases together, that Cholera more certainly kills, if let alone, and is certainly cured, if early attended to. What, then, is the earliest and almost universal symptom of approaching Cholera? I have never seen it named in print as such. During the two years above referred to, I could tell in my own office, without reading a paper, or seeing or speaking to a single person, the comparative prevalence of the disease from day to day, by the sensation which I will name, and I hope to the benefit of thousands, and perhaps not a single reader will fail to respond to the statement from his own experience. The bowels may be acting but once, or less than once, in twenty-four hours, the appetite may be good, and the sleep may be sound; but there is an unpleasant sensation in the belly—I do not, for the sake of delicacy, say "*stomach*," for it is a perversion of terms—it is not in the stomach, nor do I call it the abdomen. Many persons don't know what abdomen means. Thousands have such good health that they have no "realizing sense" of being the owners of such "*apparati*," or "*usses*," as the reader may fancy, and it is a great pleasure to me to write in such a manner that I know my reader will understand me perfectly, without having the head-ache. Who wants to hunt up dictionary words when the thermometer is a hundred at the coolest spot in his office? It is bad enough to have to write what you know, at such a Fahrenheitical elevation as I do now, but it is not endurable to be compelled to find the meaning of another by hunting over old lexicons, and, after all, running the risk of discovering that the word or phrase was, in its application, as innocent of sense as the noggin was of brains which used the expression.

Speaking then of that sensation of uneasiness, without acute pain, in the region named, it comes on more decidedly after an evacuation of the bowels. In health, this act is followed by a sense of relief or comfortableness, but when the cholera influence is in the atmosphere, even a regular passage is followed by something of this sort, but more and more decided after each action over one in twenty-four hours. The feeling is not all; there is a sense of tiredness or weariness which inclines you to take a seat; to sit down and maybe, to bend over a little, or to curl up, if on a bed. This sensation is coming cholera, and if heeded when first noticed, would save annually thousands. The patient should remain on the bed until he felt as if he wanted to get up, and as if it would be pleasurable to walk about. While observing this quiet and while swallowing lumps of ice, nothing should be eaten until there is a decided appetite, and what is eaten should be farina, or arrowroot, or tapioca, or corn-starch, or what is better than all, a mush made of rice-flour, or if preferred, common rice parched as coffee, and then boiled, as rice is usually for the table, about twelve minutes, then strain the liquid from the rice; return the rice to the stew pan and let it steam about a quarter of an hour, a short distance from the fire; it will then be done, the grains will be separate; it may then be eaten with a little butter, at intervals of five hours.

There can be no doubt that thousands upon thousands have died of cholera who might now be living had they done nothing but observed strict bodily quietness under the promptings of nature, the greatest and the best physician.

### WHAT IS "A LOOSENESS?"

An indefinite description or direction in reference to health is worse than none at all. Physicians very generally, and very greatly err in this respect, and much of their "want of success" is attributable to this very omission. A patient is told he "mustn't allow himself to become costive," mustn't eat too much, must take light suppers, mustn't over exercise. These things do much mischief. The proper way to give a medical direction is to use the most common words in their ordinary sense, and in a manner not only to make them easily understood, but impossible to be misunderstood, and to take it for granted that the person prescribed for knows nothing. How many readers of mine have an easy and complete idea of the word "expectorate" in medicine, or regeneration in religion and yet the terms expectoration and regeneration are used as glibly by preacher and physician as if their meaning were self-evident. Why shoot above people's heads and talk about justification and sanctification and glorification, and a great many other kinds of "ations," when the terms do not convey to one ear in a dozen any clear, well-defined, precise idea? And so emphatically with the words looseness and costiveness when applied to the bowels. They are relative terms, and a practical idea of what they are is only to be conveyed by telling what they are, and what they are not. One man will say he is very costive, that he has not had an action from the bowels in three or four days or more; but a failure of the bowels to act in 24 or 48 or 72 hours is not of itself costiveness, for the person may have had four or five passages in a single day; then nature requires time to make up, so as to average one a day. Costiveness applies to the hardness and dryness of the alvine evacuations, and not to relative frequency.

A more indefinite idea prevails in reference to the more important (in cholera times at least) terms looseness, loose bowels, and the like. The expression must be measured by color and consistency of the discharges in reference to cholera. We have heard and read a great deal about rice water discharges. Reader of mine, physicians, nurses, and cooks excepted, lay this down a moment, and say if you ever saw rice water in your life. Then again how is the reader to know whether the cholera rice water is applied to rice water as to color, or consistence, or taste, or smell. The term "looseness" as applied to Asiatic cholera as a premonitory symptom, is simply this: if in cholera times a man passes from his bowels even but a single time, a dirty, lightish-colored fluid, of consistence and appearance, a few feet distant, of a mixture of half and half milk and water, that is a premonition of cholera begun, and he will be dead in perhaps twenty-four hours at farthest, and as the passages become less frequent and of a darker or greener or thicker nature, there is hope of life. It does not require two such passages to make a looseness; one such is a looseness, and a very dangerous one. Nor does it require a gallon in quantity; a single tablespoonful, if it weakens, is the alarm-bell of death in cholera times.

But do not suppose that if looseness of bowels is a premonitory symptom of cholera, costiveness, that is, an action of the bowels once in every two or three days, is a preventive, or an evidence that you are in no danger; for constipation is often the forerunner of looseness. Some of the most fatal cholera cases I have seen were characterized by constipation previous to the looseness—the patient having concluded that as there was nothing like looseness, but the very reverse, he was in no danger, and consequently had no need of carefulness in eating or drinking, or anything else. Unusual constipation, that is, if the bowels during the prevalence of cholera act less frequently than usual, or if they even act with the same frequency, but the discharges are very hard or bally, then a physician should be at once consulted. That is the time when safe and simple remedies will accomplish more than the most heroic means, a few days or even a few hours later.

THEORY OF CHOLERA.

It is in its nature common diarrhœa intensified, just as yellow fever is an intensification of common bilious fever—a concentrated form of it. But what causes this loose condition of the bowels, which is not indeed a premonitory symptom of cholera but which is cholera itself?

That which precedes the loose bowels of diarrhœa and cholera is liver inaction; the liver is torpid, that is, it does not abstract the bile from the blood, or if it does, this bile instead of being discharged drop by drop from the gall bladder into the top or beginning of the intestines, where the food passes out of the stomach into the bowels proper, is retained and more or less reabsorbed and thrown into the general circulation, rendering it every hour thicker and thicker, and more and more impure and black, until at length it almost ceases to flow through the veins, just as water will very easily pass along a hose pipe or hollow tube, while mush or stirabout would do so with great difficulty; and not passing out of the veins, but still coming in, the veins are at length so much distended that the thinner portions ooze through the blood vessels. That which oozes through the bloodvessels on the inner side of the stomach and bowels, is but little more than water, and constitutes the rice water discharges, so much spoken of in this connection; that which oozes through the blood vessels on the surface constitutes the sweat which bedews the whole body shortly before death, and it is this clogging up of the thick black blood in the small veins which gives the dark blue appearance of the skin in the collapse stage.

What is the reason that the liver is torpid—does not work—does not withdraw the bile from the blood?

It is because the blood has become impure, and being thus when it enters the liver it fails to produce the natural stimulus, and thus does not wake it up to its healthful action, just as the habitual drinker of the best brandy fails to be put "in usual trim" by a "villainous article."

But how does the blood become impure? It becomes impure by there being absorbed into the circulation what some call malaria, and others call miasm. But by whatever name it may be called, this death-dealing substance is a gas arising from the combination of three substances, heat, moisture, and vegetation. Without these three things in combination there can be no "cholera atmosphere," there can be no epidemic cholera in these ages of the world. Vegetable matter decomposes at a heat of between seventy and eighty degrees, and that amount of heat in combination with moisture and some vegetable substance must always precede epidemic cholera.

The decomposition in burial grounds, in potters' fields, or of animal matter in any stage or form, does not excite or cause cholera; if anything, it prevents it. I have no disposition to argue upon these points. I merely give them as my views, which, I think, time and just observation will steadily corroborate. There are many interesting questions which might be discussed in this connection, but the article is already longer than was designed. The reader may think that he could state some strong facts in contravention of those given, but I think it quite likely that on investigation these facts of his will be corroborants. For example: how is it that cholera has raged in latitudes where snow is on the ground five or ten feet deep? The people in such countries are generally poor; myriads of them live in snow houses, which are large spaces dug in the snow, with no outlet but one for the smoke, and in this house they live with their domestic animals, and all the family offal for months together, so that in the spring of the year there is a crust of many inches of made flooring, while the interior heat from their own bodies and from the fire for cooking purposes is often eighty or ninety degrees.

### THE THEORY OF CURE.

I have said that a torpid liver is an immediate cause of cholera, that it does not work actively enough to separate the bile, the impure particles, from the blood. Whatever then wakes up the liver, removes this torpidity, or in plainer language, whatever stimulates the liver to greater activity, that is curative of cholera. Calomel is a medicine which acts upon, which stimulates the liver to action with a promptness and certainty infinitely beyond all the other remedies yet known to men, and the use of any other medicine as a substitute in any plain case of cholera, is in my opinion a trifling with human life; not that other remedies are not successful, but that this is more certain to act upon the liver than all others; and what sensible man wants to try a lesser certainty in so imminent a danger.

My whole view as to cholera and calomel is simply this, that while cholera is arrested and cured by a variety of other agents, calomel will cure in all these and thousands of others where other remedies have no more effect than a thimbleful of ashes; that calomel will cure any case of cholera which any other remedy cures, and that it will cure millions of other cases which no other remedy can reach; that when calomel fails to cure all other things will inevitably fail.

HOW DO WE KNOW ALL THIS?

The natural color of healthy and properly secreted bile is yellowish, hence that is the color of an ordinarily healthful discharge from the bowels; but as the liver becomes torpid, the bile becomes greenish, and still farther on, black. If you give calomel under such circumstances, black, green, or yellow discharges result, according to the degree of torpidity. When the liver gives out no bile at all, the passages are watery and light colored. The action of a calomel pill in cholera is to arrest the discharges from the bowels, and this it does usually within two hours, and in five, eight, or ten, or twelve hours more it starts the bowels to act again, but the substance discharged is no longer colorless and thin, but darker and thicker and less debilitating, and the patient is safe in proportion as these passages are green or dark-colored. I have seen them sometimes like clots of tar.

PREVENTIVES OF CHOLERA.

There are none, there never can be, except so far as it may be done by quietude of body and mind, by personal cleanliness, by regular and temperate habits of life, and the use of plain accustomed nourishing food.

Anything taken medicinally as a preventive of cholera will inevitably, and under all circumstances, increase the liability to an attack.

WHY?

Nothing can prevent cholera in a cholera atmosphere, beyond the natural agents of nutrition, except in proportion to its stimulating properties. The liver takes its share of the general stimulus and works with more vigor. Where the system is under the effect of the stimulus, it is safer, but it is a first truth that the stimulant sooner or later expends its force, as a drink of brandy, for example. That moment the system begins to fail, and falls as far below its natural condition as it was just before above it, and while in that condition is just as much more susceptible of cholera as it was less liable under the action of the stimulant, until by degrees it rises up to its natural equilibrium, its natural condition. You can, it is true, repeat the stimulus, but it must be done with the utmost regularity, and just at the time the effects of the previous one begins to subside. This it will at once be seen, requires a nicety of observation, and correctness of judgment which not one in a multitude can bestow, saying nothing of another nicety of judgment, that of gradually increasing the amount of the stimulant, so that the effect shall be kept up to the regular notch; for a given amount of one stimulant will inevitably fail, after a few repetitions, to produce the same amount of stimulation, and the moment that amount fails to be raised, that moment the person is more susceptible of cholera than if he had taken nothing at all.

He who takes any medicinal agent, internal or external, for the prevention of Cholera, commits an act of the most consummate folly; and I should consider myself an ignoramus or a knave were I to concoct a professed anti-cholera mixture.

### THE SUMMING UP.

When Cholera is present in any community, each person should consider himself as attacked with Cholera,

1st. If the bowels act less frequently than usual.

2d. If the bowels act oftener than twice in twenty-four hours.

3d. If the discharge from the bowels is of a dirty white in color, and watery in its consistence.

4th. If he have any indefinable sensation about the belly, which not only unpleasantly reminds him that he has such an article, but also inclines him to sit down, and makes sitting down a much more pleasant operation than usual.

Some persons may think that this fourth item is putting "too fine a point" on the matter, and that it is being over careful; but I know that these very feelings do, in a vast majority ot fatal cases of Cholera, precede the actual "looseness" so universally and so wrongfully regarded as the premonitory symptom of cholera; "looseness," is not a premonitory symptom of Cho.era.

☞ *LOOSENESS IS CHOLERA BEGUN!!*

Whenever Cholera is prevalent in any community, it is as much actual Cholera, under such circumstances, as the first little flame on the roof of a house constitutes "a house on fire."

When Cholera is present as an epidemic—as a "falling upon the people," which is the literal meaning of the word epidemic, in a liberal translation—a person may have one regular action every twenty-four hours; it may not be [illegible]ard and dry, it may not be in lumps or balls, and it may b[illegible] consistent enough to maintain its shape and form, and this [illegible] neither too costive nor too loose, and is just what it ought t[illegible] be in health; but, at the same time, if a person in a cholera atmosphere has such a passage from the bowels, and it is followed not merely by an absence of that comfortableness and sense of relief with which all are familiar in health, but by a positive sensation, not agreeable, not painful, but unpleasant, inclining to stilness, and there is a feeling as if a slight stooping or bending forward of the body would be agreeable,—these are the premonitories of Asiatic Cholera; and it is wonderful that they have never, as far as I know, been published in book or newspaper for popular information. At such a stage no physician is needed, no physic is required, only quietude on the back, ice to be eaten if there is any thirst, and no food but toasted bread, and tea of some kind, green, black, sage, sassafras, or any other of the common herbs. Keep up attention to these things until you can walk without any uncomfortableness whatever, and even feel as if it were doing you good, and until you are not sensible of anything unpleasant about the belly.

If you get tired of tea and toast, or if it is not agreeable to you, use in their place boiled rice, or sago, or tapioca, or arrow-root, or corn starch, or mush made of rice flour. With all these articles a little boiled milk may be used, or they may be eaten with a little butter, or syrup of some kind, for a change.

If, under the four circumstances named on page 4, there is not an improvement in the symptoms within a very few hours, by the three things there named, to wit:

1st. Quietude on your back, on a bed.

2d. Eating ice, if thirsty.

3d. A diet of tea and toast, or boiled rice, or some of the starches:

Then do not trifle with a holy, human life by taking any medicine on your own responsibility, nor by the advice of any unprofessional man; but, by all means, send for a physician. But if you have violent vomiting, or have a single lightish-colored, watery passage, or even a thinnish passage every hour or two, and no physician can be had in several hours, do not wait for him, but swallow a ten-grain calomel pill, and repeat it every second hour until the symptoms abate or the physician arrives; or, if at the end of two hours after the first pill has been taken, the symptoms have become aggravated, take two calomel pills of ten grains each and then patiently wait. If the passages stop, if the vomiting ceases, you are safe; and if, in addition to the cessation of vomiting, or looseness, or both, the passages become green or dark, and more consistent within eight, or ten, or twelve hours after the first pill, and, in addition, urination returns, you will get well without anything else in addition beyond judicious nursing.

The most certain indication of recovery from an attack of Asiatic Cholera is the return of free urination; for during the attack it ceases altogether,—a most important fact, but not known, perhaps, to one person in ten thousand, and is worth more than all other symptoms together.

### CAUSES OF CHOLERA.

A very great deal has been uselessly written for public perusal about the causes of Cholera. One person will tell you that a glass of soda gave him cholera, or a mess of huckleberries, or cucumbers, or green corn, or cabbages, which is just about as true as the almost universal error, that a bad cold causes consumption. A bad cold never did nor ever can originate consumption, any more than the things above named originate cholera. A bad cold excites consumption in a person whose lungs are already tuberculated, not otherwise, certainly; and so green corn, or cucumbers, or cabbages, or *any other food, whatever* it may be, which is not well digested when it passes into the stomach, will excite cholera, when a person is living in a cholera atmosphere, and the atmosphere is made "choleric" by its holding in suspension some emanation which is the product of vegetable decomposition.

### LIMESTONE WATER.

Much has been written about this agent as a cause of Cholera. Those who know least are most positive. It may be true to some extent, and, under some circumstances, it may be an excitant of Cholera; but I cannot think it is "per se"—that it is remarkably or necessarily so. It is known that the whole South-west has suffered from Cholera, New Orleans especially; yet there is scarcely a decent dwelling there which has not a cistern attached to it, *above ground*, and wholly supplied by rain water; and this is the usual drink, and it is the same case with multitudes of the better class of dwellings in the Southern country.

As to escaping prevalent Cholera, the great general rules are:

1st. Make no violent changes in your mode of life, whether in eating, or drinking, or sleeping, or exercise.

2d. Endeavor to attain composure of mind, quietude of body, regularity of all bodily habits, temperance in the use of plain, substantial, nourishing food; and let your drinks be a moderate amount of tea, and coffee, and cold water. If accustomed to use wine or brandy, or any other beverage or alcoholic stimulant, make no change, for change is death. If any change at all, it should be a regular, steady, systematic increase. But as soon as the Cholera has disappeared, drink no more.

### FRUITS, IN CHOLERA TIMES,

Are beneficial, if properly used. They should be ripe, raw, fresh, perfect,—should be eaten alone without cream or sugar, and without fluids of any kind for an hour after, and they should not be eaten later in the day than the usual dinner hour of two P. M.

In Cholera times, nothing should be taken after dinner, except a piece of cold bread and butter, and a cup of tea of some kind. This, indeed, ought to be the rule for all who wish to live long and healthfully.

The indefinite unpleasantness in the bowels, which I have so much insisted upon as the real premonitory symptom of

Asiatic Cholera begun, whether there be looseness or constipation, most probably precedes every acknowledged attack of Cholera, from hours up to days. There are no means for proving this, certainly; for the mass of people are too unobserving. But it most certainly is a safe rule in cholera times, to regard it as a premonitory, and to act accordingly.

Whatever I have said of Cholera in the preceding pages, I wish to be understood as applicable to what has come under my own observation during the general prevalence of Cholera in a community.

In different States and countries there are circumstances which modify the disease, its symptoms, and everything connected with it, such as locality, variety of exciting causes, their different degrees of virulence or concentratedness, the different habits and modes of life. These things constitute the reason of the various modes of treatment, and the great error has been the publishing of a successful remedy in one locality, and relying upon it in another. But the treatment by quietude, ice, and calomel, is equally applicable on every spot of the earth's surface, wherever a case of Epidemic Cholera occurs, since the essential cause of Cholera is everywhere the same, to wit, the miasm of vegetable decomposition, the effects of that cause are the same, to wit, a failure on the part of the liver to work with sufficient vigor to withdraw the bile from the blood and pass it out of the system; and the mode of removing that effect is the same, to wit, the stimulation of the liver to increased action. And although, in milder forms, a variety of agencies may stimulate the liver to work, and thus restore health, yet inasmuch as calomel is infinitely more reliable than all other liver stimulants yet known, it is recommended as having precedence of all others, on the ground previously named, that when danger is imminent and a few hours makes the difference between life and death, it is unwise to trust to a less certain agent when the more certain one is equally at hand and is the easiest medicine known to be taken, as it has no appreciable taste, its bulk is exceedingly small, and by reason of its weight it sinks to the bottom of the stomach and cannot be rejected except in rare instances.

Some of my views are peculiar, perhaps. They were formed from observations made in 1832, '3 and '4, my first experiences being on board a crowded steamboat which left Louisville, Kentucky, in October, 1832. In twenty-four hours the cholera broke out. It had just reached the west from Canada. No one knew anything about its nature, symptoms, or treatment, practically, and the panic was terrible. I had retired early A Virginia gentleman was lying on the floor suffering from a attack. At midnight I awoke and found the cabin deserted, not a living creature in it, nor on the boat either, as well as I now remember, and every berth but mine was entirely divested of its bedding. The man had died, and they were airing the boat, while a few were engaged in depositing him at the foot of a tree in a coarse wooden box, on the banks of the Ohio. The boat was bound for St. Louis, but few of her passengers to that port, or officers, lived to reach their destination. I was young then, had perfect health, and knew no fear. Ever since that terrible "trip," and the experiences of the following years, everything that I have seen or read on the subject of cholera has seemed to me to confirm the views advanced in the preceding pages, and I trust that general readers, as well as professional men, who may chance to see this article, will hereafter direct their attention to all facts bearing upon cholera, and notice how far such observed facts will bear them out in concluding, 1st, that epidemic Asiatic cholera cannot exist aside from moisture, heat, and vegetable matter; 2d, that quietude, ice, and calomel will cure where anything else will, and will succeed in multitudes of cases where all things else have signally failed.

CALOMEL PREJUDICES.

If, then, calomel is such an admirable agent in cholera, why is it not universally used? I might as well ask, if honesty is the best policy, why are not the majority of men honest from principle? It is because men are ignorant or misinformed. Many persons do not know the power of calomel in curing cholera, while others are afraid of it because it sometimes salivates. Suppose it does—better to run the risk of salivation than to die. And even if salivated, a man is not necessarily permanently injured by salivation. I have been badly salivated several times very many years ago, but I believe I have as good health as most men. I do not recollect to have lost three meals from sickness in fifteen years past, except from sea sickness, and no doubt there are tens of thousands of persons who have been salivated can speak similarly. But the objection is perfectly childish when it is remembered that perhaps a thousand persons in succession may take calomel and not two in the thousand be salivated. I might say not two in ten thousand, and that in a vast majority of those who are not designedly salivated, this salivation is the result of injudicious administration; thus,

Salivation is caused by keeping the system too long under the influence of calomel, in two ways:

1st, By giving small doses at short intervals.

2d, By giving an amount so small that it fails to work itself off in ten or twelve hours.

3d, By giving a larger amount, but mixing opium in some form or other with it; for in all cases the more opium or other anodyne you give with a dose of calomel, the longer it will be in producing its legitimate action.

The best method of administering calomel is to give enough at one time to make it act of itself within twelve hours, and if it does not act within that time, take an injection of half a pint of tepid water, or of a tablespoonful of salts in a half pint of warm water every hour until the bowels do act. Any action of the bowels at all after six hours since taking the calomel may be set down as an action from calomel, and nothing need be done to "work it off."

If salivation is not designed, it is not best to give a dose of calomel oftener than once a week.

By observing the two rules just stated, I do not believe that any general practitioner will have one case of undesired salivation in ten years practice.

It is important for the reader to remember that there are sporadic cases, that is, scattering cases of cholera which may not be preceded by a constipation, or looseness of bowels, or uneasiness sufficiently decided to have attracted the observation of the patient; for in many cases the patient declares that he "*felt*" as well as he ever did in his life, or acquaintances remark that he "*appeared*" to be in perfect health, and yet to-day he is dead of cholera. Yet, I very much doubt if a case of cholera ever occurred without the premonitions above named in a greater or less degree. Still, for all practical purposes, and to be on the safe side, let no one who has looseness to-day in cholera times, conclude that it cannot be cholera, because he "felt" so and so the day before, or because no premonitions were observed; rather let him conclude they were slight or unobserved, and act as he should do if he were perfectly assured that he had at that moment in his own person, undisputed epidemic Asiatic cholera. The truth is, it is as impossible for a man in perfect health to be stricken down in a moment with this dangerous disease, as it is for a man who has been honest from principle for a lifetime, to become in a day a forger or a swindler.

As far as my observation has extended, I believe that the most frequent of all exciting causes of cholera is going to bed too soon after a hearty meal, whether it be a late dinner or merely a supper of fruits and cream or milk, with sugar. I think that eating freely of fruits or berries, ripe, raw, and perfect, with any fluid after them, and then going to bed in an hour or two, will excite cholera in cholera times. I am inclined to think that huckleberries with cream or milk, except in very small quantity, make a dangerous dish in cholera times.

It may subserve a good purpose to remark that I have written on this subject not to support a theory, but to draw attention to the suggestions, and least of all to obtain a cholera practice. I never treated a cholera case except gratuitously. I do not visit persons out of my office, except in rare cases. I prescribe only for those who come to see me and who write to me, and my practice is closely confined to ailments of the throat and lungs, and has been for ten or fifteen years.

I will close the subject with answering an inquiry which no doubt has occurred to the reader as a conclusive refutation of all that I have said as to the fundamental cause of cholera, to wit:

If cholera is the result of heat, moisture, and vegetable matter in combination, why has it not prevailed from time immemorial? Because the climates of the world, and of the various countries of the earth, the constitutions, and habits of life, and modes of living are constantly changing; hence new diseases are making their appearance from time to time, while others have vanished from the world. And when a single element of many is changed, an entire new combination may be the result. But whatever may be that new or changed element, it can no more, as far as our present knowledge extends, excite epidemic cholera without the aid of vegetable decomposition, than powder can be ignited without the aid of fire.

### CONCLUDING OBSERVATIONS.

While Cholera prevails, no marked change should be made as to the general habits of a regular temperate life—as long as the person feels entirely well—but the moment the great premonitory symptom is observed even in a slight degree, to wit, *an indefinable uncomfortableness in the belly, inclining to rest,* then an instantaneous change should be made from physical activity to bodily rest—from mental activity to mental relaxation—from the habitual use of wines, or malt, or other alcoholic drinks to total abstinence—from everything of the kind; using ice or ice-water as a substitute, or cold spring water, a few swallows only in any twenty minutes; but if ice is to be had, and there is thirst, it may be eaten continuously from morning until night.

Whatever may have been the diet before, it should be changed at once to tea and toast, or cold bread and butter, with plain meat, salted or fresh, whichever is relished most—I mean that these changes should be made on the first appearance of *belly-uncomfortableness,* and if in six or eight hours you are not decidedly better, send for a physician. If you are better, continue your own treatment until the feeling in the belly has entirely disappeared and you have a desire to walk about, and experience a decided relief in doing so.

If you have over two (or three at most) passages within twenty-four hours, do not make an experiment on your life by taking even a calomel pill, simple as it is, unless it be wholly impracticable to obtain a physician within three or four hours.

### DIET IN CHOLERA TIMES.

If you have no special liking for one thing more than another, and have not even the premonitory symptom, to wit, the *belly-uneasiness,* then the following diet will render you more secure:

Breakfast.—A single cup of weak coffee or tea, with toasted bread, or cold bread and butter, and a small piece of salt meat, ham, beef, fish, or the like, *and nothing else.* Dinner—Cold bread, roasted or broiled fresh meat of some kind, potatoes, rice, hominy, samp, or thickened gruel. For Dessert—Rice, or bread pudding, or sago, arrow root, tapioca, farina, corn starch, prepared in the usual manner, *and nothing else fluid or solid.* Tea, or Supper—A single cup of weak tea of some kind, or coffee, with cold bread and butter—*nothing else.*

Eat nothing between meals; go to bed at a regular early hour, not later than ten o'clock; attend to your business with great moderation, avoiding hurry, bustle, worriment of mind; wear thin woollen flannel next the body during the day, air it well at night, sleeping in a common cotton night garment; remain in bed of mornings, after you have waked up, until you feel rested in all your limbs; but do not by any means take a second nap. Do not sleep a moment in the day time, and let all your enjoyments and recreations be in great moderation.

Fruits have not been named, because it is so difficult to get them fresh, ripe, perfect—many looking so, are wormy. Except potatoes, no vegetables are named, because they more readily sour on the stomach, require more power of digestion, while they do not afford as much nutriment and strength to the body in proportion

The preceding article on Cholera was first published in the JOURNAL for August, 1854, soon after which, letters came from different and distant parts of the country, expressing the belief of the writers that its suggestions were instrumental in saving their lives; it seemed to commend itself to the common-sense of almost every reader, and hence was copied by the press more extensively than was any article we had ever written on any subject. Some papers copied it entire. The *Scalpel*, whose talented editor is not over-given to the praise of any body or any thing, said in his August number, page 519, that it was "a most excellent article on Cholera; but we don't understand the calomel; all the rest is admirable." Our views were simply these: that no medicine should be taken in any case, unless it was impracticable to obtain a physician; in that event, rather than do nothing and die, it was the simplest, safest, easiest, and best plan to swallow ten grains of calomel, made into a pill with any kind of gum-water or mucilage, for the several reasons:

1. It could be almost always found close at hand;

2. It would stay on the stomach when nothing else would; and,

3. It would sooner, more certainly, and with greater safety arrest the looseness (and thus keep the disease at bay until a physician could be had) than any other known remedy, and would be more likely to cure the disease without any thing else being done beyond quiet, ice, and a woolen flannel compress around the abdomen. The *Scalpel* mistook us to mean that a person should wholly rely on the calomel, while we wished it to be regarded simply as *the safest, surest, and most accessible arrester of the progress of cholera until a physician could be had.* In most cases under our own observation, in Southern and Western practice, no other medicine was needed; but the watchful care of a good physician was imperative, every two hours, until the patient was out of danger. Dr. Reese, of the *New-York Medical Gazette*, said of the same article: "It is timely, and, in the main, judicious. Dr. Hall brings to the subject professional and practical knowledge, and writes with much ability. We recommend this number as one which will amply repay perusal, there being so much of truth and good sense on the subject. In very many cases, there could be no better practice; but other and more potent means are often required. Dr. Hall's mode of treatment is greatly to be preferred to any other we have seen in the newspapers, where we have observed numerous 'cures,' so called, which, if used, will be disastrous and fatal." As Dixon and Reese were among the very best medical scholars and writers of the time, it may be rationally concluded that the article was truthful, practical, and safe; and as no new facts as to the nature, symptoms, and treatment of cholera have been established in the last twelve years, and as the disease in 1865 has presented no new phases, it may perhaps be safe to infer that what we wrote in 1854 would, a dozen years later, be applicable to the disease, should it reäppear during the coming spring and summer. If it is a very cold summer, it can not appear; if it be a very hot and dry summer, it will not appear in the North, but will ravage the whole South; if it be an alternation of rains and hot suns, it will devastate the whole country, sweeping off its hundreds of thousands where filth, feebleness, and fear prevail, leaving intact those who, having good health and brave hearts, live temperately and regularly. This is our theory; we will not stop to explain or defend.

During 1865, the cholera, in its march westward toward our shores, has exhibited an unusually malignant type. Two thousand died daily in Constantinople, and this may be the measure of its malignity with us; and it certainly behooves all to look at it understandingly and with a high moral courage. This, with regularity, temperance, and cheerfulness, will be a shield against its ravages everywhere, not only as to individuals, but as to whole communities; but it must be a cleanliness in person and in habitation, so as to secure the breathing of a pure atmosphere day and night.

But as there are some who will not be willing to take calomel in case of an attack, and as it may not be obtainable, it is well to know of some other means less objectionable to the prejudice, or one which has been used and tried by intelligent persons in thousands of instances, and always with reported success when its use has been commenced when an unusual looseness of the bowels first manifested itself. It was the favorite remedy of the missionaries during the recent ravages of the disease in Asia, and as it seemed to be adapted to the type of the disease on its present march, we may infer that it will be as available in this country as it has been in the East. But even this remedy should not be used if a physician can be had, and neither this nor any other remedy should be employed ten minutes longer than the securement of a physician.

The reason for this injunction, which is intended to be spe cial and personal to each reader, is, that we can not calculate certainly that the disease will put on precisely the same phase in any two localities, or during any two consecutive months or even weeks; and as the physicians are in the midst of it all the time, and are necessarily close observers, their own experience will quickly detect differences, and their skill and judgment will make an application adapted to the ever-varying circumstances. It is therefore advised that every family have prepared for themselves, by their own family physician, or by the druggist or apothecary with whom they are best acquainted, four ounces, that is, eight table-spoons, of the following: Equal parts of Laudanum, Spirits of Camphor, and Tincture of Rhubarb. A grown person should take from thirty to sixty drops as soon as the diarrhea begins, and repeat the dose at each succeeding action of the bowels until they cease to move. Sixty drops make a tea-spoonful, so that to save time in counting the drops, measure it by a tea-spoon, as ten or fifteen drops, more or less, is not of much consequence. It would be a good precaution, and be safer, as it saves time, to increase each succeeding dose by ten drops until the discharges cease, and then take the mixture at intervals of four hours, after the passages have ceased, diminishing each dose by twenty drops. If the stomach will not retain it, put a mustard plaster about ten inches square over the region of the stomach, and keep repeating the dose until it is retained. This mustard plaster should be made by mixing good kitchen mustard with strong vinegar, making it of a pasty consistence, so that it can be taken up and spread with a knife. Let the plaster remain as long as it can be well borne; but in order to prevent its taking off the skin and making an ugly or troublesome sore, let a very thin piece of muslin or paper wetted interpose between the mustard and the skin.

If the disease has been allowed to run on so long that the above remedy does not have a decided effect within two or three hours, and if a physician can not be had, take from half to a whole tea-spoon of a mixture of equal parts of Tincture of

Opium, Tincture of Capsicum, Tincture of Cardamom Seed, and Tincture of Gingerberry. If still no physician can be had, and the person has passed into the cold stage, or stage of collapse, when the skin is cold and blue and bedewed with a clammy sweat, then do what the missionaries testify has saved many:

1. Let two or three persons dip flannels in hot rum and rub the limbs.
2. Apply bottles of hot water to the armpits and feet.
3. Give half a table-spoon of brandy every fifteen minutes, and continue all this as long as there is any hope of life.

Let it be understood that we have no experience of the value of these remedies in the different stages of cholera in this country; but as the missionaries report it very successful in Turkey during the ravages of 1865, it is advised here, in all cases where physicians or experienced nurses can not be had.

We here close this article with a piece of advice not hitherto published, but which will save many a life if the cholera does appear next summer: "If the bowels have been accustomed to act soon after breakfast, and you are called to the privy before breakfast, or you wake up very thirsty, not being a liquor-drinker, you will have the cholera before night unless you adopt the following precautions:

1. Send for a physician.
2. Remain warm in bed.
3. Bandage the abdomen tightly with stout woolen flannel, at least a foot broad.
4. Eat nothing, unless hungry, but boiled rice with boiled milk.
5. Drink nothing; but if thirsty, chew and swallow bits of ice abundantly.
6. Remain in bed with a calm and fearless mind, (for you can't die under the circumstances,) until you feel as if it would do you good to get up and take a walk; and if the walking gives no feeling of weariness or desire to sit down, you are a well man.

